# Nitrogenous Derivatives of Phosphorus and the Origins of Life: Plausible Prebiotic Phosphorylating Agents in Water

**DOI:** 10.3390/life7030032

**Published:** 2017-07-29

**Authors:** Megha Karki, Clémentine Gibard, Subhendu Bhowmik, Ramanarayanan Krishnamurthy

**Affiliations:** Department of Chemistry, The Scripps Research Institute, 10550 North Torrey Pines Road, La Jolla, CA 92037 USA; meghak@scripps.edu (M.K.); cgibard@scripps.edu (C.G.); sbhowmik@scripps.edu (S.B.)

**Keywords:** prebiotic phosphorylation, nitrogen-phosphorus derivatives, phosphoramidates, (di)amidophosphate, origins of life

## Abstract

Phosphorylation under plausible prebiotic conditions continues to be one of the defining issues for the role of phosphorus in the origins of life processes. In this review, we cover the reactions of alternative forms of phosphate, specifically the nitrogenous versions of phosphate (and other forms of reduced phosphorus species) from a prebiotic, synthetic organic and biochemistry perspective. The ease with which such amidophosphates or phosphoramidate derivatives phosphorylate a wide variety of substrates suggests that alternative forms of phosphate could have played a role in overcoming the “phosphorylation in water problem”. We submit that serious consideration should be given to the search for primordial sources of nitrogenous versions of phosphate and other versions of phosphorus.

## 1. Introduction

One of the most important elements for life on the earth is phosphorus [1]. As a critical component in the hereditary material and metabolites in extant life, phosphorus (P) has attracted the attention of the origin of life and astrobiology scientific community [2,3,4]. The origin and reactivity of phosphorus in the form of minerals, organophosphates, inorganic (poly)phosphates, and various derivatives has been investigated intensively to resolve the quintessential ‘prebiotic phosphorylation in water problem’ [2]. The focus has been predominantly on P–O species such as inorganic orthophosphates, condensed phosphates like trimetaphosphate (TMP) or polyphosphates as prebiotic phosphorylating reagents [3]. Phosphorylation of prebiotic organic molecules in aqueous environment faces two problems: (a) the water molecule itself can and will react with the phosphorylating agent, thus hydrolyzing the phosphorylating reagent and rendering it ineffective, and (b) in some cases the phosphorylation process involves the formation and removal of a water molecule, which in an aqueous environment becomes thermodynamically unfavorable [2]. In this context, the ‘phosphorylation problem’ with inorganic phosphate sources under a variety of low water activity conditions has been reviewed recently [4].

However, there is a parallel approach where “alternative forms” of phosphate have been considered inspired by the discovery of phosphonic acids in Murchison meteorite by Schwartz [5,6]. The occurrence of phosphite derivatives in the archean marine carbonates also suggests that the primary source of these reduced phosphorus (P) species in the early Earth might have been meteoritic [7,8]. The pioneering work of Lauretta and Pasek [9,10], and Kee [11] has been leading this front. This approach of considering alternative forms of P, could be further broadened to survey a wider spectrum of phosphorus P–O derivatives such as compounds containing P–N bonds as plausible prebiotic reagents. Towards that end, in this review, we document the reactions of nitrogenous derivatives of phosphate, from the prebiotic-, synthetic organic- and bio-chemistry perspectives, and propose that such P–N derivatives could be considered to provide plausible (alternative) prebiotic solutions to the ‘phosphorylation in water problem’.

We begin with the prebiotically plausible amidophosphate derivatives which have been shown to be excellent phosphorylating reagents in aqueous medium, of biogenic and prebiological organic molecules, in a prebiotic context. Then, we describe other nitrogenous analogs (P–N derivatives) of various oxidized states of phosphorus that have been discovered and used in other contexts (such as organic synthesis) along with their useful applications in origins of life chemistry research. In the next section, biochemically relevant P–N derivatives that are used as phosphorylating (phosphoryl transfer) agents are discussed. We then present a survey of plausible prebiotic P–N based species and end with a perspective that the reactivity of the plausible prebiotic, synthetic and biological P–N variants may provide inspiration and insights for finding a library of solutions to the ‘prebiotic phosphorylation in water problem’.

## 2. The Development of Amidophosphate as a Potential Prebiotic Phosphorylating Reagent

In pursuit of activated prebiotic phosphorylating compounds, trimetaphosphate has long been considered as one of the promising and potential candidate for phosphorylation in aqueous medium [12,13,14,15,16,17,18,19,20,21,22], even though the availability of polyphosphates in the primitive earth is believed to be scarce [23]. The presence of pyrophosphate and tripolyphosphate in volcanic magma outflows [24], and their detection in reaction mixture of apatite with basalt at higher temperature seems to be in line with the earlier prediction by Griffith et al. that apatite could be a source of P_4_O_10_ at elevated temperature [25]. The presence of the inorganic pyrophosphate was also demonstrated by Baltscheffsky et al. as the major product of photophosphorylation obtained by isolated chromatophores from *Rhodospirillum rubrum* [26]. Kornberg and co-workers have also shown various examples of the enzymatic synthesis of inorganic phosphates [27,28,29] and the use of enzymatically synthesized polyphosphates for the conversion of ADP to ATP [30], and has hypothesized about the role of polyphosphates in prebiotic chemistry [31].

**Figure 1:**
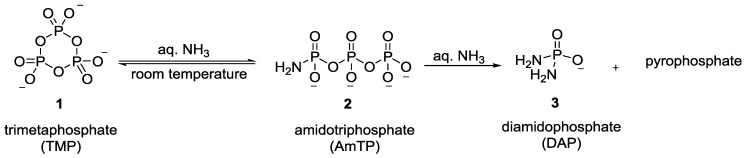
Formation of amidotriphosphate (AmTP) **2** and diamidophosphate (DAP) **3** as described by Quimby and Flautt [35] and Feldman and Thilo [36] starting from trimetaphosphate (sodium salt) **1** and aqueous ammonia.

The ineffectiveness of trimetaphosphate as a reagent for the phosphorylation of glycolaldehyde in aqueous solution for the formation of glycolaldehyde phosphate (a molecule that was important in the context of the pyranose-RNA oligonucleotides [32,33]), led Krishnamurthy et al. to explore other variants that could be derived from trimetaphosphate [34]. Inspired by the reports by Quimby et al. [35] and Feldmann et al. [36]*,* who showed that ammonia reacts efficiently with trimetaphosphate **1** in water to give rise to amidotriphosphate (AmTP) **2** (Figure 1), Krishnamurthy et al. investigated the potential of AmTP to phosphorylate glycolaldehyde [34].

**Figure 2:**
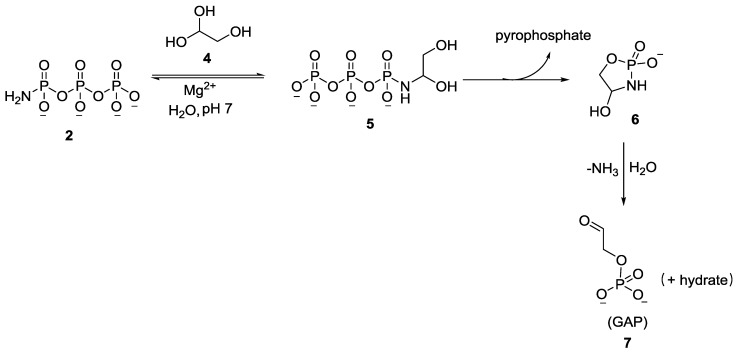
Phosphorylation of glycolaldehyde to yield glycolaldehyde phosphate (GAP, **7**) using amidotriphosphate (AmTP, **2**) as shown by Eschenmoser and co-workers [34].

**Figure 3:**
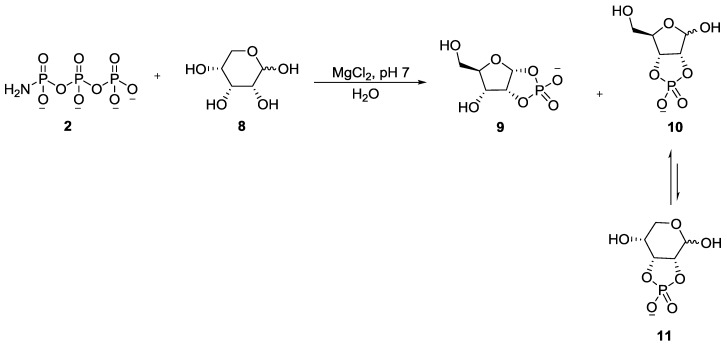
Regioselective intramolecular phosphorylation of sugars using AmTP **2** in aqueous medium, exemplified here by the phosphorylation of ribose **8** [37].

It was shown that glycolaldehyde **4** was phosphorylated by AmTP **2**, in the presence of divalent metals in water at room temperature and near neutral pH, to form glycolaldehyde phosphate **7** (GAP) in quantitative yields (Figure 2). The reaction was effective even at micromolar concentrations of both reactants which is attributed to (a) the enhanced electrophilicity of the carbonyl group in **4**, (b) the excellent nucleophilicity of the amine group in **2** and (c) importantly, the intramolecular transfer of the phosphate group [34]. The combination of these factors effectively overcomes the “water problem”, which has been (and continues to be) a major stumbling block for phosphorylation in aqueous medium [4]. Subsequently, in a follow-up work by the same group, AmTP **2** was shown to phosphorylate a library of α-hydroxy aldehydes (aldoses such as ribose **8** and other sugars) in aqueous medium (Figure 3) [37].

**Figure 4:**
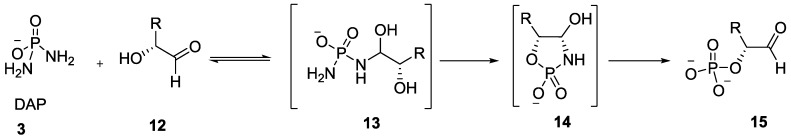
DAP mediated intramolecular phosphorylation of aldoses in a regioselective manner. R = CH_2_OH and (CHOH)_n_–CH_2_OH [37].

In this context, the use of diamidophosphate (DAP) **3** as an effective replacement of AmTP **2** for the phosphorylation in water was discovered [37]. DAP **3**, contains P–N bonds, and was previously shown by Feldman and Thilo to be produced by the further ammonolysis of AmTP **2** (Figure 1) [36]. Krishnamurthy et al. in 2000 showed the ability of DAP **3** to phosphorylate aldoses (glyceraldehyde, tetroses and pentoses) in aqueous medium. The reaction takes place via the attack of one of the nucleophilic NH_2_ groups of DAP **3** on the electrophilic carbonyl group of α-hydroxy aldehydes followed by an intramolecular attack of the α-hydroxy group on the amidophosphate intermediate **13** (with the other protonated NH_2_ group acting as the leaving group). This yields the cyclic phosphoramidate intermediates **14**, identical to the ones that were obtained with the AmTP **2** as the reagent. Hydrolysis of the cyclic intermediate **14** leads to the 2-phosphate-derivatives **15** of aldoses in a regioselective fashion with excellent conversion yields (Figure 4) [37]. All the reactions with sugars and the phosphorylating reagents **2** and **3**, take place in a single-pot scenario with high conversions. The reactions take place over days to weeks with no discernible side reactions, because the rate of hydrolysis of the phosphorylating reagents is much slower when compared to the rate of phosphorylation reactions.

**Figure 5:**
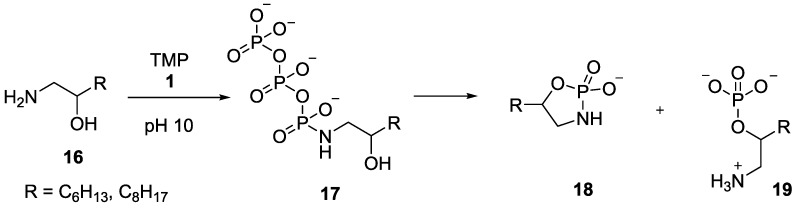
Trimetaphosphate (TMP) **1** mediated phosphorylation of α-hydroxy-*n*-alkylamines **16**, further illustrating the utility of the P–N derivatives towards phosphorylation in aqueous medium by intramolecular phosphate transfer [38].

This principle of intramolecular phosphoryl-transfer by the use of amidophosphates as phosphorylating reagents in a prebiotic context, was further investigated by Sutherland and co-workers. They demonstrated that the phosphorylation reaction of long chain β-hydroxy-*n*-alkylamines **16** with trimetaphosphate **1** generated the corresponding *N*-triphosphate **17**, which underwent an intramolecular phosphorylation to form potentially prebiotic amphiphiles such as *O*-monophosphate **19** in the absence of divalent metals at pH 10 (Figure 5). The reaction presumably proceeds via the formation and hydrolysis of the cyclophosphoramidate **18** (not observed). Depending on the length of the alkyl chain, varieties of different products were obtained, and the longest chain compound, where R=C_8_H_17_ produced *O*-monophosphate product **19** [38].

**Figure 6:**
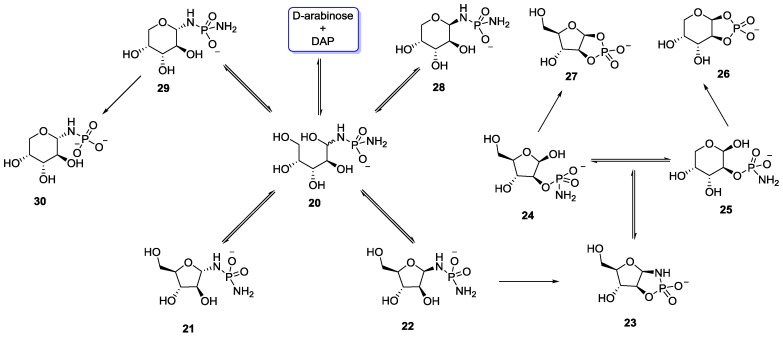
Phosphorylation of arabinose with DAP **3** in aqueous medium demonstrated by Sutherland and co-workers [39].

The Sutherland group, while investigating the potentially prebiotic synthesis of nucleotides via arabinose-3-phosphate in the presence of cyanamide, observed that direct phosphorylation of arabinose couldn’t be achieved. They showed that when arabinose was treated with DAP **3**, it formed some proportion of 1,2-cyclic phosphoramidate **23** and its cyclophosphate analogue **27** along with other phosphorylated species (Figure 6) [39]. However, no formation of the desired arabinose-3-phosphate was observed (unlike the phosphorylated product obtained with d-ribose on reacting with DAP), demonstrating the necessity of the *cis*-disposition of the adjacent hydroxyl groups for intramolecular five-membered ring mediated phosphoryl transfer.

**Figure 7:**
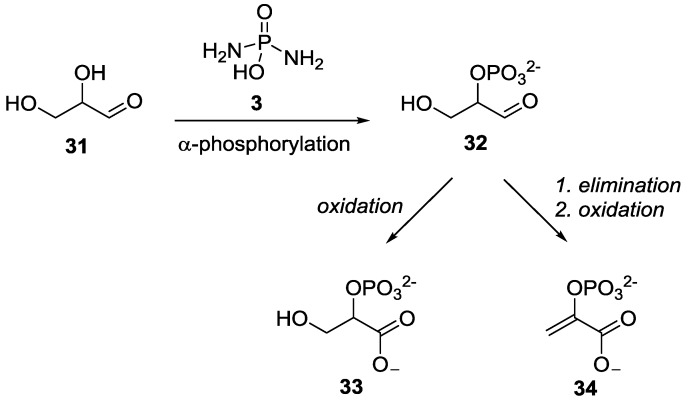
Powner’s work on regioselective α-phosphorylation of glyceraldehyde **31** with DAP **3** further leading to glycolysis intermediates [40].

More recently, Powner and co-workers demonstrated the use of DAP **3** for the potential prebiotic synthesis of phosphoenol pyruvate **34** which is the highest-energy molecule involved in extant metabolic pathways of glycolysis [40]. Building on the previous work on the regioselective phosphorylation of glyceraldehyde **31** with DAP **3** using magnesium salts [34], Powner et al. showed that indeed the same result is obtained at neutral pH, but by using a phosphate buffer (obviating the need for periodic pH adjustment), to yield glyceraldehyde-2-phosphate **32**. The thus generated glyceraldehyde-2-phosphate **32** was converted to glycerate **33** and phosphoenol pyruvate **34** by subsequent manipulations (Figure 7).

## 3. Other P–N Derivatives and Their Chemistries 

From the above examples, it becomes clear that by using the nitrogenous versions of phosphate, the phosphorylation of prebiotic substrates can be achieved even under aqueous conditions. Therefore, as an alternate to using phosphate itself, the use of its P–N derivatives provides another way to approach the “prebiotic phosphorylation in water problem”. There are plenty of other examples where P–N derivatives (synthesized or formed in situ) have been shown to be relevant in the context of prebiotic chemistry and origins of life studies. This section includes an overview of the history and the development of these and related P–N derivatives.

## 4. Formation of *N*-Phosphoryl Amino Acids and Their Reactivity: Condensation of Amino Acid and (Poly, Cyclic)Phosphates

**Figure 8:**
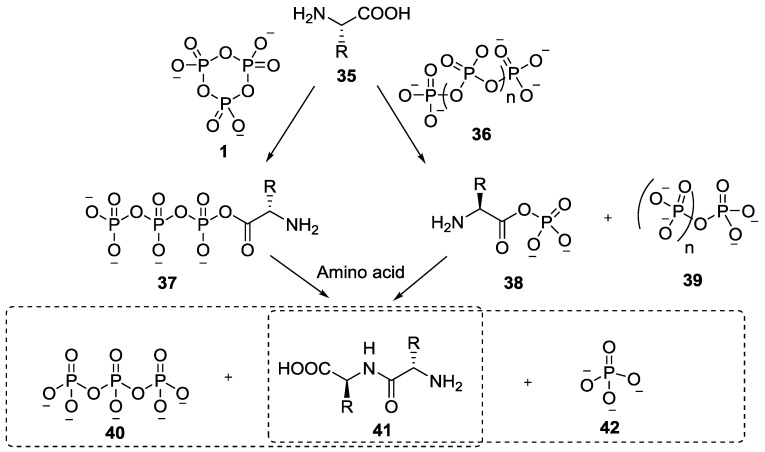
Rabinowitz’s mechanism for the reaction of trimetaphosphate **1** or polyphosphates (sodium salts) and amino acids leading to the formation of dipeptide **41** [41,42].

In 1969, Rabinowitz showed that by reacting trimetaphosphate **1** or other linear polyphosphates (sodium pyrophosphate, tripolyphosphate, polyphosphate or ammonium polyphosphate) with glycine at pH 7–8, diglycine was formed in good yield (best yield up to 36% was obtained with sodium trimetaphosphate at 70 °C; or at room temperature up to 31%) [41,42]. The mechanism was proposed to involve the attack of the carboxylic acid group (of the amino acid) on the polyphosphates leading to an activated acylphosphate intermediate **37** and **38** (Figure 8), which are subsequently attacked by the amino group (of another amino acid) to give peptides **41**.

**Figure 9:**
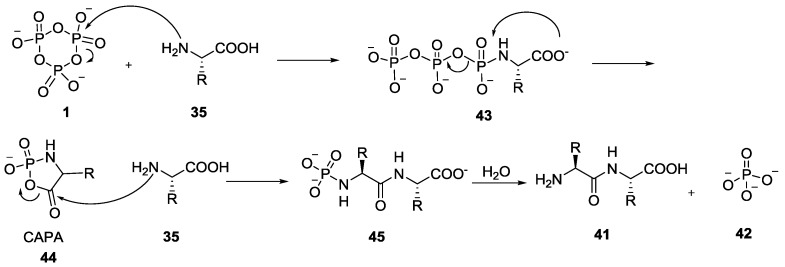
Orgel’s mechanism for dipeptide formation via cyclic and phosphoryl-activated amino acids (CAPA) intermediate **44** involving the attack of the amino group on the trimetaphosphate **1** [44].

Feldmann and Thilo also reported that ammonia and alkylamines can react with trimetaphosphate **1** to form open chain phosphoramidates, with the process being reversible in acidic solution [36,43]. In the early 1970s, Orgel et al. suggested that the carboxylic acids were almost unreactive with trimetaphosphate **1** under the conditions where the dipeptide was formed [44]. They proposed a different mechanism involving the nucleophilic attack of the -NH_2_ group of the amino acid onto the phosphorus of trimetaphosphate leading to an acyclic phosphoramidate intermediate **43** (Figure 9) [44]. Surprisingly, the cyclic and phosphoryl-activated amino acids (also known as cyclic acyl-phosphoramidates or CAPA **44**) remained relatively unexplored for quite some time after its discovery.

**Figure 10:**
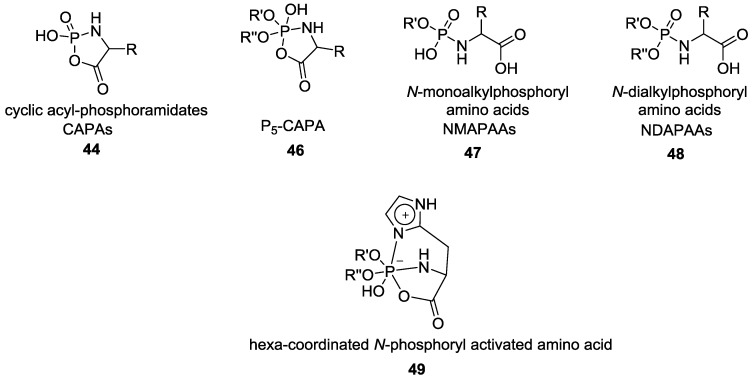
Derivatives of CAPA **44**, **46** and acyclic *N*-alkylated phosphoryl amino acids intermediates **47, 48** and hexa-coordinated P–N intermediate **49** observed during the formation of small peptide mediated by phosphate species as explained by Yu et al [49].

In early 1990, Zhao et al. extensively studied the dialkyl derivatives of CAPAs **44** (Figure 10). For those new intermediates, it was proposed that the formation of a pentacoordinated phosphorus center was responsible for the activation of the carboxylic acid that can further react with a free amine of another amino acid residue (the same reactivity as previously described by Orgel was observed). However, this process involving the participation of the phosphoryl group that could provide some insight for the prebiotic synthesis of peptide (or protein) faced a major solubility problem. For example, the *N*-dialkylphosphorylated amino acids are insoluble in water and therefore the coupling reactions have been described in organic solvent such as methanol, phenol, butanol or chloroform [45,46]. The same group later proposed that an even more reactive hexa-coordinated P–N intermediate **49** could have led to the formation of longer peptides under aqueous conditions (Figure 10).

An alternative prebiologically plausible formation of *N*-monoalkylphosphoryl amino acids (NMAPAAs, Figure 10) **47** was described starting with the reaction of CAPAs in a mixture of methanol/water at pH 11. *N*-mono-methoxyphosphoryl glycine was obtained with a yield of 88% after recrystallization [47,48]. 

**Figure 11:**
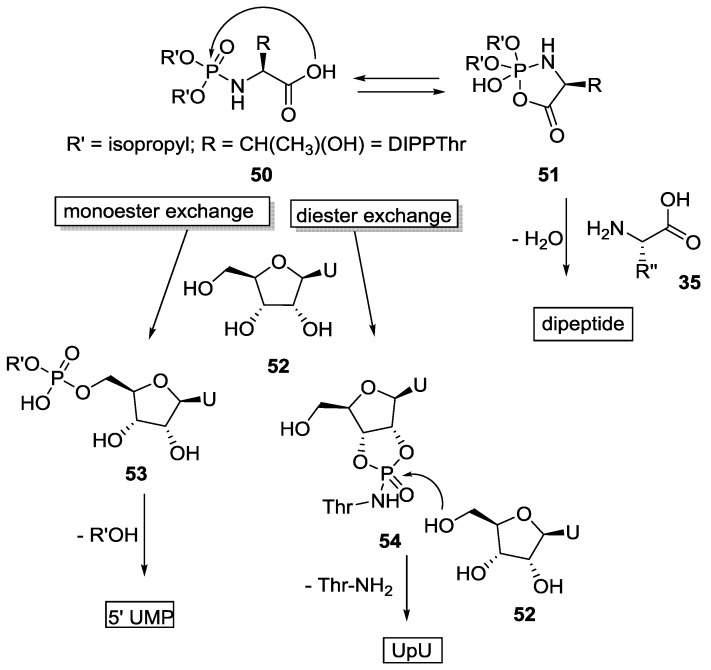
Dual electrophilic centers (carbonyl and phosphoryl) present in α-CAPAs **51** leading to the formation of dipeptide by the attack of an amino acid **35** at the carbonyl center and the formation of (oligo) nucleotides via the attack of nucleoside **52** at the phosphoryl center **50** respectively. (5'UMP—5' uridine monophosphate; UpU—3',5'-uridyluridine; Thr–NH_2_—threonine).

The reactivity of dual electrophilic centers present in α-CAPAs as determined by isotopic analysis (^18^O, ^15^N) was highlighted in another publication by Zhao et al. [47]. The corresponding penta-coordinated *N*-dialkyl amino acid cyclic phosphates (P5-CAPAs) **46** could react both at the carbonyl and phosphorus center depending on the nature of the substrate [47,49,50]. The pathway involving the attack of a nucleophilic amine at the activated carbonyl center has already been shown (Orgel and Rabinowitz [44]) to produce short peptides. The alternative reaction of a hydroxyl positioned directly at the phosphorus center led to the formation 5'-UMP or UpU. Zhao et al*.* studied the formation of homopeptide from *N*-(*O,O*-diisopropyl) phosphothreonine (DIPPThr) in the presence of each of the four nucleosides (adenosine, uridine, cytidine and guanosine) at room temperature [50]. They showed that while cytidine and uridine increased the yield of the homopeptide (up to tetramer, < 8% overall), adenosine had no effect and guanosine was detrimental [50]. The fast atom bombardment mass spectrometry (FAB-MS) and fast protein liquid chromatography (FPLC) profile also showed the simultaneous formation of 5'-NMP as well as dinucleotides (UpU and (Up)_2_) when uridine, cytidine or adenosine were used (Figure 11). Though the reaction required anhydrous pyridine [51], the finding suggests that a connection between the formation of peptides and oligopeptides via the formation of a phosphoramidate was possible, and pointed the need to move to plausibly prebiotic conditions.

## 5. Implementation of P–N Nucleoside Phosphoramidate Derivatives

**Figure 12:**
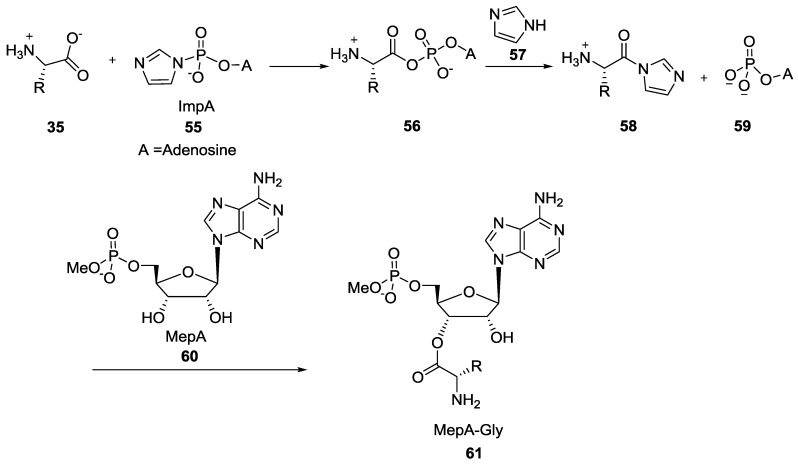
Introduction of imidazole 5'-adenosine monoamidophosphate (ImpA) **55** for the activation of amino acid residues to form longer peptides as demonstrated by Orgel et al. [52,53].

In the late 70s, Orgel et al. published several studies regarding the formation of dipeptide where the first amino acid was attached via the carboxylic moiety to the 2'(3')-hydroxyl of a 5'-methyl phosphate adenosine (MepA-Gly **61**, Figure 12) by the reaction of the –COOH part of the amino acid with the phosphoroimidazolide intermediate of adenosine (ImpA) **55** leading to the activation of the carboxylic acid that could further react with the free hydroxyl of the ribonucleoside [52,53]. However, this water-soluble intermediate **61** mostly led to the formation of diketopiperazine.

**Figure 13:**
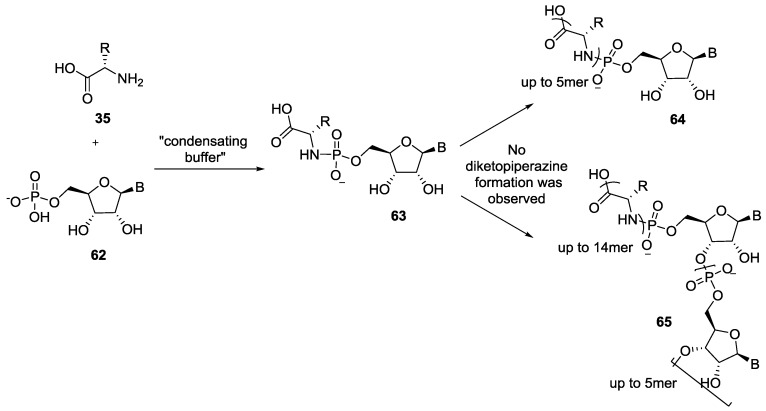
The condensation of an amino acid **35** and a 5'-nucleoside monophosphate **62** in a condensing buffer leading to the simultaneous formation of oligonucleotides and oligopeptides amino acid 5'-nucleoside amidophosphate [54,55,56].

Recently Richert et al. published several studies showing that peptides up to 14 mers can be formed when the first amino acid residue was attached to the 5'-phosphate of a nucleotide through a P–N bond [54]. Contrary to Orgel’s strategy, in this case the peptide formation took place from the N_term_ to the C_term_ and avoided the formation of diketopiperazine, which inhibits the oligomerization. The Richert group used “condensating buffers” containing *N*-(3-dimethylaminopropyl)-*N*′-ethylcarbodiimide hydrochloride (EDC), carbonyl diimidazole (CDI) or cyanamide to couple the first amino acid with nucleotides to form the corresponding phosphoramidate **65** (Figure 13) [54,55,56]. They also observed the formation of oligonucleotides **65** indicating a parallel phosphorylation of nucleotides. These results show the advantage and importance of the strategy of involving P–N derivatives for phosphoryl transfer, and should provide further inspiration for the search of more prebiotically plausible reagents and conditions.

**Figure 14:**
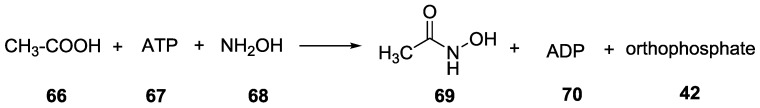
The activation of the carboxylic acid with ATP **67** (without an enzyme) through the formation of an acetyl phosphate, trapped by hydroxylamine **68** leading to the formation of acetylhydroxamate **69** [57].

Another nucleotide-phosphorylating agent is ATP **67**, which is the most common energy currency in biology; but whether ATP **67** was present and utilized as a prebiotic phosphorylating reagent is not known. In 1961, Schatz et al. showed that ATP **67** can transfer a phosphoryl group without the presence of an enzyme, but requires the presence of a divalent metal [57]. They concluded that carboxylic acids **66** such as acetate or glycine could act as acceptors instead of orthophosphate. When the reaction was performed in the presence of hydroxylamine **68**, acetylhydroxamate **69** was formed by “trapping” acetyl phosphate (Figure 14). It was also proposed that aminoacyl phosphates could have reacted with amino acids instead of hydroxylamine leading to the formation of peptides instead of hydroxamates.

**Figure 15:**
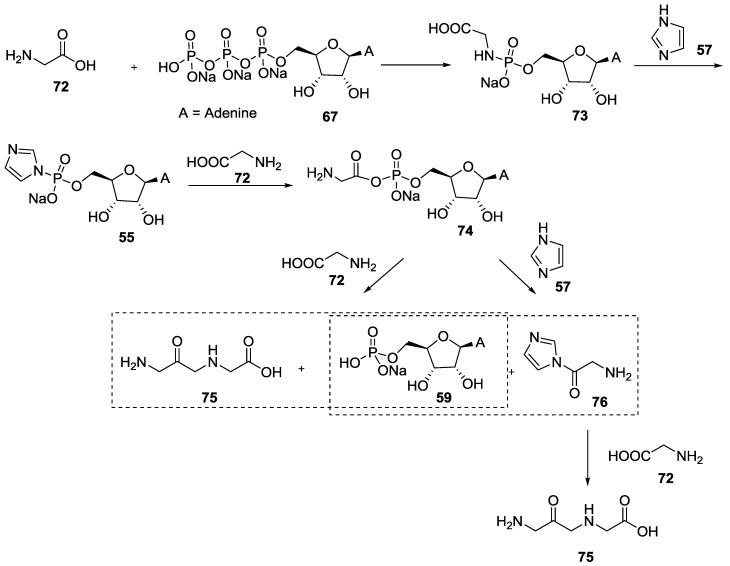
Formation of diglycine **75** through the condensation reaction between –NH_2_ of glycine **72** with ATP **67**. Imidazole **57** was required for the condensation to occur due to the formation of the amidophosphate intermediate **55** [58].

Inspired by these findings, Orgel et al. examined the reactivity of various amines with ATP **67** in the presence of magnesium chloride [58]. They found that ammonia, glycine, ethylenediamine or imidazole can react with ATP **67** in a dry state to form phosphoramidate if enough magnesium cations were present. Regarding the CAPAs formation scenario through an intramolecular pathway, Orgel proposed that another kind of activation for the prebiotic synthesis of peptide could be possible, involving the formation of P–N intermediates, as outlined in Figure 15.

The formation of a phosphoroimidazolide intermediate of adenosine **55** (ImpA) was considered critical for the reaction to proceed. Warming up the phosphoramidate **73** alone did not lead to dipeptide formation, while the same reaction in presence of one equivalent of imidazole **57** allowed the formation of diglycine **75** in up to 10% yield.

## 6. Use of Phosphoroimidazolides for the Polymerization of Nucleotides in the Origin of Life Context

The transmission of hereditary information is achieved by the replication of nucleic acid in contemporary organisms (enabled by enzymes), and it is believed that a similar/corresponding abiotic replication process may have played a crucial role in the origins of life. In 1974, Orgel and Lohrmann established a list of prebiotic acceptable “rules” in the context of the research toward the abiotic replication of nucleic acid [59]. It was proposed that water must be the only solvent used, and that the starting building blocks should be derivable from simple gases like methane, carbon monoxide, ammonia or nitrogen for example. They studied several potential prebiotic water-soluble activators for nucleotide condensation, like carbodiimides, and as mentioned previously, they were particularly interested in imidazolide derivatives.

The 5'-phosphoroimidazolides of nucleosides proved to be good activated species for the formation of oligonucleotides. Numerous studies have shown their use with or without template [60] with various divalent metals [61], self-condensate or reacting with other nucleotides [62]. In 1974, Orgel et al. described that the small amounts of the dinucleotides could be obtained from nucleoside phosphoramidates. These P–N containing phosphoramidates were derived from polyamines containing at least three amine groups that are strongly adsorbed by sodium and magnesium montmorillonite clays under dilute conditions [63].

Phosphoroimidazolide of various nucleosides, particularly of adenosine, ImpA **55** has been widely used for prebiotic oligonucleotide synthesis with or without the presence of a template [64]. Orgel and Lohrmann studied the reaction of the *N*-phosphoroimidazolides of the dinucleotides pUpG and pCpA in presence of appropriate templates. For example, GpGpUpG was formed by the condensation of ImpUpG with GpG on a poly(C,A) template containing nearly 6 cytidine residues for each adenosine. However, the yield was always low and in some cases no product was formed at all [60]. Recently, Szostak et al. have provided insights regarding the mechanism of the hydrolysis of ImpA **55** at the phosphorus center by both experimental and computational studies [65].

**Figure 16:**
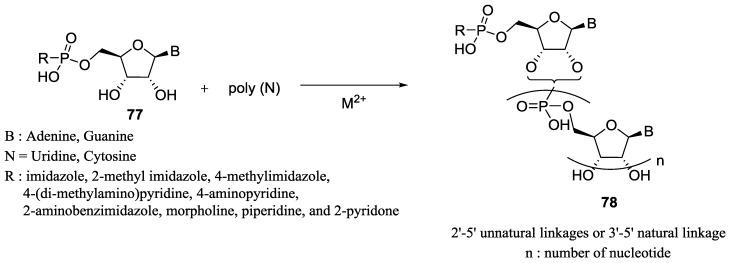
A summary of the different 5'-phosphoramidate (P–N) species that was pioneered by Orgel [68] and expanded to other variants by Ferris [69,70,71] and Szostak [72] (to cite a few) for non-enzymatic oligonucleotide poly(N)-template mediated replication studies. The resulting product oligonucleotide formed by those methodologies contained 2'-5' non-natural linkage and/or the natural 3'–5' phosphodiester linkage.

In 1975, Sawai and Orgel showed that the divalent zinc cation could catalyze the formation of oligonucleotides from nucleosides phosphoroimidazolides in water without a template [66]. When ImpA **55** was reacted in water in the presence of Zn^2+^, oligoadenylic acid was formed up to 25.2% yield. ImpApA was rapidly formed and disappeared slowly leading to the accumulation of pApA and pApApA. It was also shown that Zn^2+^ enhanced the formation of the phosphodiester bond by a factor of 10, disfavoring the hydrolysis of ImpA **55** at the same time but led to 76–90% of the 2'–5' unwanted linkages [66]. A year later, Sawai studied the effect of several divalent metals for the catalysis of the oligomerization of the phosphoramidate ImpA in water and showed that Pb^2+^ afforded up to 57% of condensate product. It was later found that Co^2+^, Zn^2+^, Mn^2+^ or Ni^2+^ could also promote the synthesis of the phosphodiester bond efficiently and, regardless of the cation used, 2'-5' linkages were predominantly formed [67]. A predominance (75%) of natural 3'–5' linkage was obtained when ImpA **55** was condensed on a poly(U) template in the presence of Pb^2+^ as a catalyst. The reaction was efficient with a conversion of up to 35% for the formation of oligomers containing 5 or more nucleotide units. In 1981, Inoue and Orgel found that the 2-methylimidazole phosphoramidate of guanosine (MeImpG) could polymerize on a poly(C) template leading predominantly to the natural 3'–5' linkage **78** without the need of a divalent metal (Figure 16). Substantial amounts of long oligomers (up to 50 mer) were formed with up to 89% conversion of the starting phosphoramidate at 0 °C after 14 days [68].

Ferris et al. extensively studied the mineral-mediated oligomerization of ImpA [69,70]. They systematically replaced the imidazole moiety by other nitrogenous heterocycles and studied the reactivity of the resulting 5'-amidophosphate of adenosine. They observed that 4-(di-methylamino)-pyridine, 4-aminopyridine, 2-aminobenzimidazole, morpholine, piperidine, and 2-pyridone could be reacted with 5'AMP in water in the presence of l-ethyl-3-(3-(dimethylamino)-propyl)-carbodiimide (EDAC) at pH 5 (Figure 16). Since this coupling reagent is not believed to be prebiogically relevant and the activated adenosine phosphoroimidazolide needs to be purified on the resin prior to the coupling reaction [71], it provides further impetus to search for more plausible prebiotic versions and conditions in the context of origin of life research. More recently, Szostak has improved on the phosphoroimidazolide chemistry by using 2-aminoimidazole in place of 2-methylimidazole, and has demonstrated a marked advantage in the template mediated non-enzymatic copying and replication [72]. The wide use of activation by phosphoroimidazolide nucleotides highlights the importance of P–N chemistries and points to the next challenge: how can this phosphoroimidazolide species be generated and regenerated in a plausible prebiotic context.

Recently, McGown et al. showed the formation of the phosphoroimidazolide ImpN (N= adenosine, guanosine, cytidine or uridine) directly in the same aqueous buffer in which the oligomerization reactions occur without the need of prior purification of the phosphoramidate intermediate. This in situ activation led to the formation of up to 10 mers with montmorillonite clay catalyst and oligonucleotides up to the tetramer without the clay catalyst [73]. Like many other researchers before in this field, they used EDC as a non-prebiotic *in situ* coupling reagent (as a proxy for the more prebiotically relevant carbodiimide), based on the premise that carbodiimide could be formed by the isomerization of cyanamide under prebiotically plausible conditions [74,75].

## 7. Role of P–N Species in Oligonucleotide Chemistry

**Figure 17:**
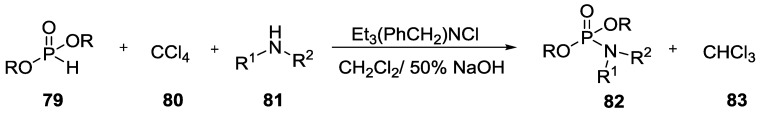
Atherton, Openshaw and Todd's pioneer works demonstrating the synthesis of phosphoramidate **82** via reaction of dialkylphosphite **79** and alkylamine **81** [77].

P–N derivatives have also found use in the application in synthetic methodology of nucleotide chemistry. In 1954, Atherton and Todd described methods for the phosphorylation of amines; and since then, the chemistry they developed have attracted the scientists in the field to comprehend the mechanistic details, conditions, scope and applications of these methodologies [76]. The Atherton-Todd (AT) reaction was initially applied for the synthesis of phosphoramidates **82** and phosphoramidate hydrazides by reacting dialkyl phosphite **79** with alkyl amine **81** in the presence of a non-polar solvent (Figure 17) [77].

**Figure 18:**
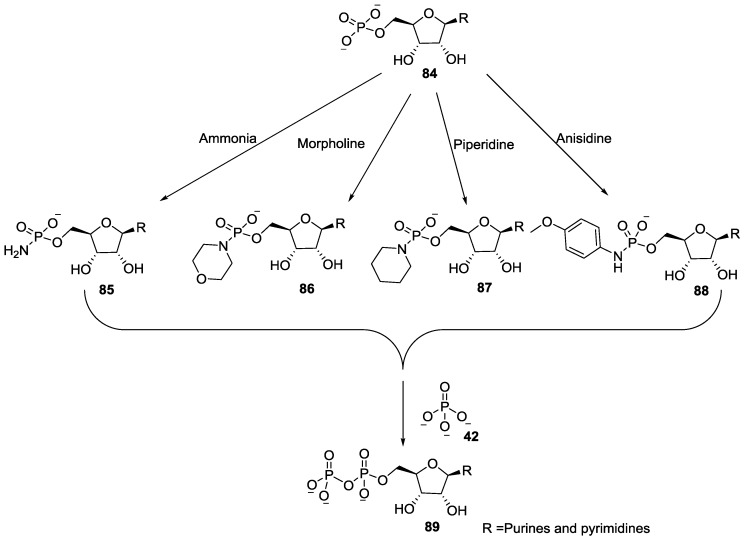
Synthesis of nucleoside-5'-diphosphates **89** with various phosphoramidate species described by Moffatt and Khorana [78].

Later, Khorana introduced a concept of ‘activation of nucleoside’ for the synthesis of long oligonucleotides by reacting phosphoramidate species which was generated by the reaction of nucleoside-5'-phosphate with dicyclohexylcarbodiimide followed by its coupling with the desired nucleoside. The first pragmatic application of nucleoside-5'-phosphoramidates such as nucleoside-5'-phosphoromorpholidate **86**, phosphoropiperidate **87** and phosphoroanisidate **88** was for the synthesis nucleoside-5'-diphosphates **89** (Figure 18) and triphosphates [78].

**Figure 19:**
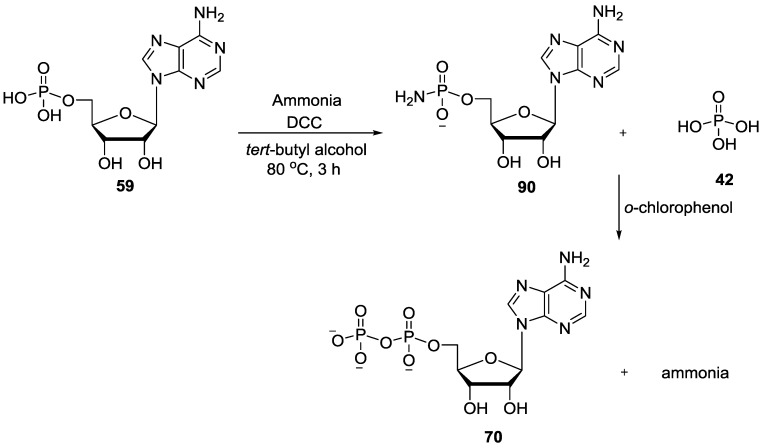
Formation of adenosine 5'-diphosphate **70** by the reaction of adenosine 5'-phosphoramidates **90** with orthophosphoric acid via displacement of NH_3_ as the leaving group [79].

Khorana and Todd showed that amidophosphates derived from nucleosides can be used also for generating nucleotides such as di- and triphosphates. The adenosine 5'-phosphoramidates **90** were conveniently prepared by the condensation of readily available adenosine 5'-phosphate **59** with ammonia in the presence of an excess of dicyclohexyl carbodiimide followed by the reaction with orthophosphoric acid **42** in *o*-chlorophenol to give adenosine 5'-diphosphate **70** by displacing NH_3_ in moderate yields [79] (Figure 19).

**Figure 20:**
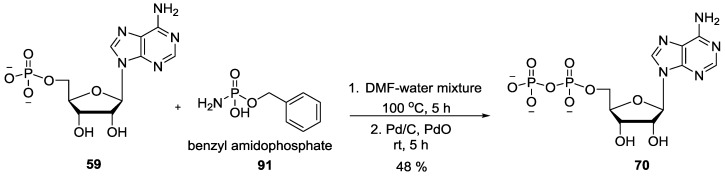
Convenient method for the formation of adenosine 5'-pyrophosphate **70** on reaction of monobenzylesters of amidophosphate **91** with adenosine 5'-monophosphate **59** [80].

Similarly, the monobenzyl esters of amidophosphates **91** were observed to behave as the selective acylating agents for phosphoric acid anions in the presence of free alcoholic hydroxyl groups to phosphorylate nucleoside 5'-phosphate to nucleoside 5'-pyrophosphate and nucleoside 5'-triphosphate in good yields [80] as shown in Figure 20 with adenosine 5'-monophosphate **59** as an example.

An improvement for the oligonucleotide synthesis over the phosphodiester method was introduced by Letsinger with his phosphotriester method [81], which was later modified by Caruthers’s phosphoramidite method which played a pivotal role for the routine synthesis of oligonucleotides [82].

**Figure 21:**
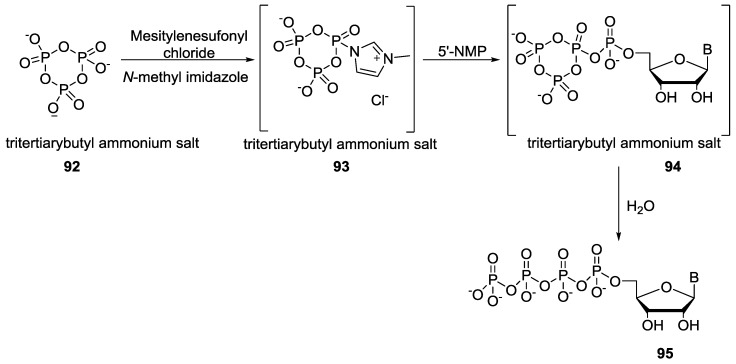
Synthesis of nucleoside tetraphosphate **95** via the formation of activated P–N compound **93** as demonstrated by Taylor and co-workers [83].

Mohamady and Taylor demonstrated an efficient route for the formation of nucleotide tetraphosphate or pentaphosphate by using the trimetaphosphate as the phosphorylating agent. The trisodium salt of the trimetaphosphate was converted to the corresponding tritertiarybutyl ammonium salt **92** to solubilize the trimetaphosphate in organic solvent (acetonitrile). Then, the phosphorus center of tritertiarybutyl ammonium salt of trimetaphosphate **92** was activated by treating it with mesitylene chloride and *N*-methylimidazole to form intermediate **94** which contains the more reactive P-imidazole center followed by its reaction with NMP to give the corresponding nucleoside tetraphosphate **95** (Figure 21) [83].

**Figure 22:**
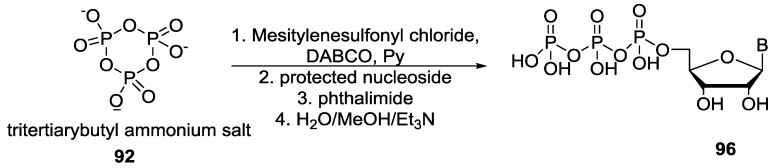
Synthesis of nucleoside triphosphate **96** by the activation of trimetaphosphate with DABCO [84].

The same group again reported an analogous technique to synthesize nucleoside triphosphate (NTP) **96** by the reaction of tritertiarybutyl ammonium salt of trimetaphosphate **92** with 1,4-diazabicyclo[2.2.2]octane (DABCO), mesitylenesulfonyl chloride (MsCl) and phthalimide although the role of phthalimide in that reaction is uncertain [84]. Both methods used organic solvent for the phosphorylation reactions (Figure 22).

**Figure 23:**
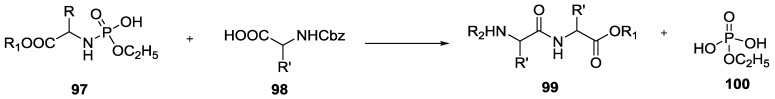
Peptide formation reaction using preformed amidophosphate of an amino acid **97** with another *N*-protected amino acid **98** [85].

Amidophosphates have also been used for short peptide formation from a synthetic view point. In 1958, G. Schramm and H. Wissmann observed that amino acid residues can be activated through the condensation of the amine moiety with polyphosphate. Phosphorus pentoxide was partially dissolved in an alcoholic solvent and was reacted with a C-protected amino acid to form the corresponding amidophosphate **97**. This activated amino acid then reacted with the carboxylic acid of an *N*-protected amino acid **98** to form peptides **99** (Figure 23) [85].

## 8. Miscellaneous Application of Compounds Containing P–N Bonds

**Figure 24:**
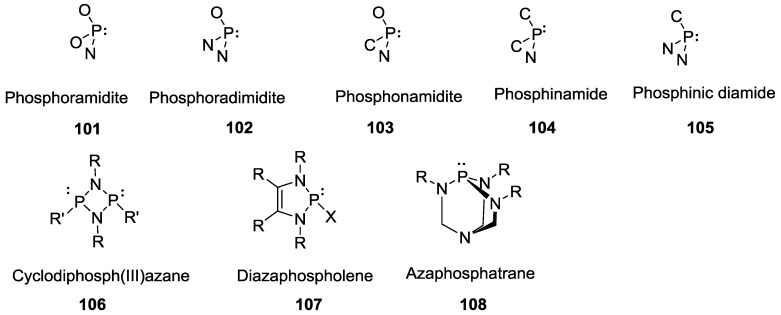
Various other types of P–N bond linkages known in the literature.

Compounds containing P–N bonds have been extensively studied due to their physiological (selective toxicity) and catalytic properties such as *N*-phosphorylated carbamates [86]. Phosphines containing P–N bonds are known as amino phosphines which are another class of compounds that exhibits versatile reactivity owing to its phosphorus (III)-nitrogen system. Phosphazenes and phosphazanes [87] contain formal P–N double bonds and single bonds respectively; these are further classified into sub-types based on the coordination number of the phosphorus. Figure 24 gives an overview of the class of these compounds and the readers are referred to the literature cited above for further details.

**Figure 25:**
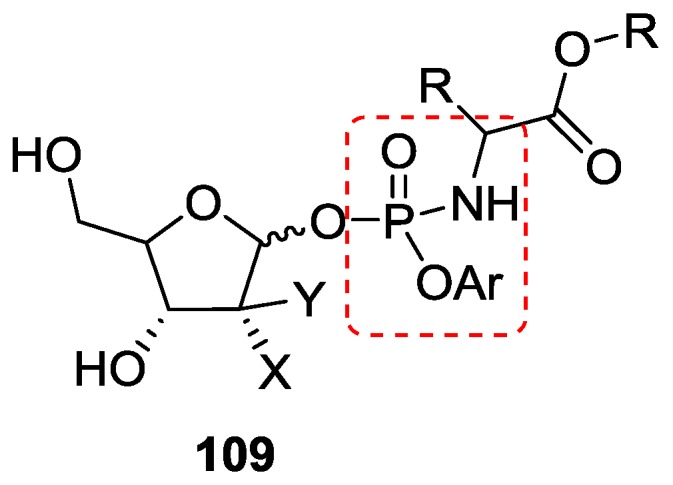
Phosphoramidate ProTides of (fluoro)-deoxyribose [89].

The structures containing covalent P–N bonds as in phosphoramidates are ubiquitous in flame proof materials [88] and chemical fertilizers. McGuigan et al. synthesized and evaluated the phosphoramidate ProTides of (fluoro) deoxyribose which are used as an antiviral prodrug **109** (Figure 25) [89].

**Figure 26:**
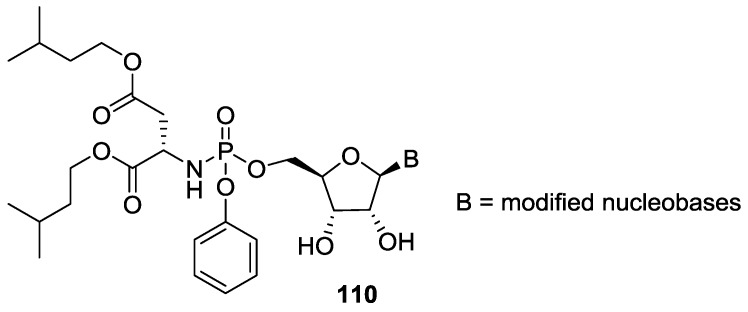
Protide libraries with modified nucleobases [91].

The utility of synthetic phosphoramidates as prodrugs is important in synthetic biology. The synthesis of (alkoxy/aryloxy)-phosphoramidates was achieved by the treatment of phosphorochloridate with *N*-methyl-imidazole (NMI) as was shown by Schinazi and co-workers [90]. Gao et al. has also recently used (aryloxy)phosphoramidate ribose derivatives as the building blocks and synthesized libraries of nucleobase modified protides (Figure 26) [91].

**Figure 27:**
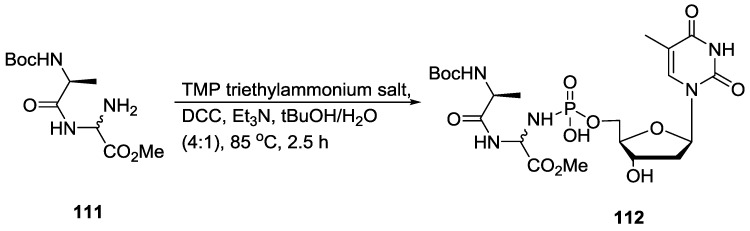
Herdewijn’s work on the synthesis of 5'-peptidylnucleotides which can be used as pronucleotides [92].

Herdewijn and co-workers also developed P–N containing 5'-peptidyl nucleoside monophosphate analogues **112** by incorporating phosphoaminal, -hemiaminal or -hemithioaminal functionalities using a coupling reaction between dipeptides and phosphate or phosphorothioate moieties (Figure 27) [92]. These peptidyl nucleoside monophosphates have been shown to act as pronucleotides for the enzymatic synthesis of xenobiotic informational systems.

## 9. P–N Phosphorylation in Biology

**Figure 28:**
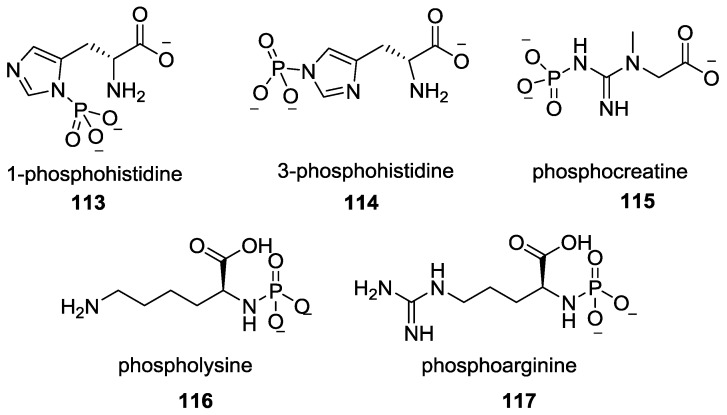
P–N bonds containing species that are used in extant biochemical pathways.

In biology, phosphorylation plays a pivotal role in signal transduction, energy transfer and functional regulation [93,94]. A process called *N*-phosphorylation of proteins that occurs on His, Arg, and Lys forms P–N bond to obtain phosphohistidines **113** and **114**, phospholysine **116** and phosphoarginine **117** (Figure 28) leads to the generation of high-energy species which act as phosphoryl group transfers and also serve as intermediates in the enzymatic catalysis. The significance of biological P–N species has been given equal stature as P-O species in cell functioning [95,96]. The role and contribution of the P–N bonds in modern biology is highlighted by the progress in understanding the cell-signaling pathways via *N*-phosphorylation of protein. A new signal transduction pathway was identified using histidine phosphorylation of P-selectin upon invigoration of human platelets [97].

**Figure 29:**
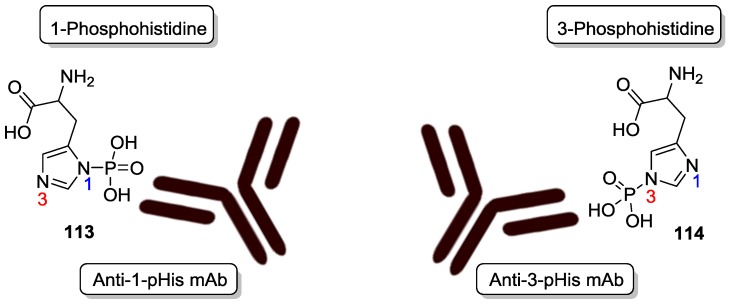
Monoclonal 1- and 3-phosphohistidine antibodies.

To study the role of histidine phosphorylation (pHis) in mammalian signaling, which was previously been under explored due to the lability of the phosphoramidate (P–N) bond and the lack of phosphohistidine-specific antibodies, Hunter et al. developed monoclonal 1- and 3-phosphohistidine antibodies as novel reagents for histidine phosphorylations (Figure 29) [98].

**Figure 30:**
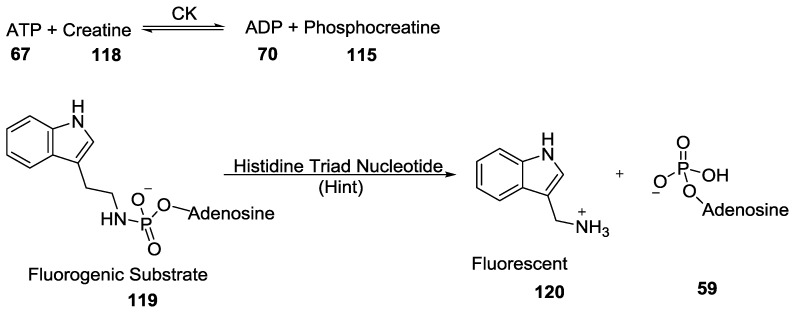
(Top) The generation of phosphocreatine which is a phosphoryl transfer agent and (below) Human and Escherichia coli Histidine Triad Nucleotide Binding Proteins (Hint) activated phosphoramidate pronucleotide as described by Wagner et al. [101].

Another important phosphorylating reagent in biology, phosphocreatine **115** (PCr, a guanidinophosphate) was discovered in skeletal muscle [99] and serves as an intracellular energy reserve by acting as a phosphoryl transfer agent (mediated by creatine kinase, CK) leading to the production of ADP **70** to regenerate ATP **67** and creatine **118** as shown in the following reaction in Figure 30 [99,100].

Wagner et al. have done a comparative study of the phosphoramidase substrate specificity of Human and *Escherichia coli* Histidine Triad Nucleotide Binding Proteins (Hint) using phosphoramidate pronucleotides and have shown that the therapeutic utility of nucleoside phosphoramidates could be expanded by improving their cellular uptake and also by incorporating an additional tissue targeting compound (Figure 30) [101].

## 10. Plausible P–N Sources in a Prebiotic Context?

The various P–N examples considered above in the plausible prebiotic, synthetic and biotic perspectives raise the compelling question as to whether there can be reasonable and plausible primordial sources of P–N that could have been available. There have been plenty of studies of plausible prebiotic P–O sources as pointed out in the introduction (and see also the discussion below). However, there seems to have been no necessity (from a prebiotic perspective) to consider corresponding P–N analogs, even though P–N chemistries have been invoked in origins of life studies as exemplified by the central role played by the development of phosphoroimidazolides of nucleotides necessary for the RNA world hypothesis. And the reason seems to be solely based on the focus on the role of phosphates in extant biological pathways. However, as will be argued below, there may be other compelling reasons to reconsider this approach.

The first reason is that primordial P–N sources are indeed available. That it is not unreasonable to propose primordial P–N containing compounds gains some credibility based on the fact that the first series of phosphorus compounds that were detected in the interstellar medium (ISM) diffuse clouds and several star-forming regions were in fact P–N species [102], whose original discovery was based on suggestions from thermodynamic equilibrium modeling [103]. For example, Sutton et al. observed a weak feature of P–N near the frequency of the *J* = 5–4 transition during the analysis of Orion-KL spectral line [104]. To verify the existence of interstellar P–N and to determine the nature of its chemical environment Ziurys searched for the *J* = 2–1, 3–2, 5–4 and 6–5 rotational transitions of this species in molecular sources and found all the four lines toward Orion-KL [105]. Their analysis suggested the presence of phosphorus nitride in the “plateau” or “doughnut” region as hot dense gas and the large abundance of phosphorus nitride suggested that the high temperature processes are responsible for the synthesis of P–N in Orion. Turner and Bally also identified the P–N compounds in the Orion (KL) interstellar medium [106]. Furthermore, Rivilla et al. reported that abundances of P–N exceeded that of P–O in evolving stars [107]. However, finding P–N species in interstellar space (while encouraging) needs to be translated to the practical and actual availability of P–N compounds on/in the (exo)planet, for which there seems to be little evidence (perhaps because of conversions of P–N compounds to other species) based on simulations [108]. And in that regard, there has been not much documentation in the literature so far, primarily because the focus has been phosphate and related P–O containing compounds.

The challenges concerning the prebiotic phosphorylation by phosphate containing minerals [4,5,6,109,110] could provide impetus for another set of possible prebiotic phosphorylating sources—the P–N analogs, and leads to our hypothesis that prebiotic chemistry could have used a different and more reactive form of phosphate such as P–N derivatives. For example, the study of phosphate and/or reduced P–O containing minerals could be used as a guide to look for reasonable P–N containing terrestrial sources. In that spirit, we review here shortly the corrosion studies of reduced P minerals, and the reaction of phosphate-minerals in eutectic solvents, with the hope that they may provide a basis for speculations as to whether consideration of such “alternative solutions” could be extended to nitrogen containing solvents, environments and atmospheres that could give rise to P–N species capable of phosphorylation in aqueous medium.

**Figure 31:**
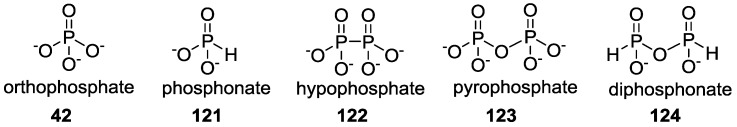
Representative structures of different P species observed by the aqueous corrosion of Fe_3_P.

The formation of reduced state P compounds from the meteorite mineral schreibersite, a common mineral in iron meteorites was demonstrated by Lauretta and Pasek [8,9] and Kee [111,112,113,114]. The corrosion of iron phosphide (Fe_3_P), an analogue of schreibersite, in water containing acetate gave diverse water soluble reduced phosphate species such as orthophosphate, hypophosphate, phosphites, and even organic phosphate compounds such as cyclic organic phosphate, phosphonoacetate and others [10] (Figure 31). The redox reactions taking place in water containing acetate and Fe_3_P were proposed to be responsible for the formation of such reduced phosphate from the minerals.

But their inability in producing the predicted phosphorus species while re-investigation led them to propose a radical based mechanism for the formation of the phosphorus compounds during such corrosion. Their proposed mechanism involves generation, propagation and recombination of different radicals to explain the formation of the phosphorus salts and phosphorylated organic compounds during such corrosion [9].

The corrosion of schreibersite, (Fe,Ni)_3_P, under prebiotic condition was also demonstrated by Bryant et al [114]. They further showed that in the presence of photochemical irradiation, H-phosphinic acid, a reduced phosphorus derivative, dominates during the corrosion and such form of phosphorus derivatives were more soluble to produce higher order phosphates. They showed that the approximate corrosion rate of Fe_3_P in saline solution was about 0.2% per week to about 10% per year [114], which suggested that meteoritic phosphides released reduced phosphorus on geologically short time scales. They also showed that H-phosphinic acid and pyruvate (a prebiotic molecule) were reacted in water to form cyclic phosphorus compounds. The H-phosphinate in the system reacts with pyruvic acid to form a di-insertion product which forms amide linkage with an amine [115].

An alternate route to synthesize a wide range of organophosphates of biological significance in a deep eutectic solvent (2:1 urea and choline chloride), utilizing various orthophosphate sources was again demonstrated by Gull et al. [116]*.* As opposed to the suggestions made in the paper [116], the activation of phosphates or phosphites is likely by the attack of the oxygen of the urea on the phosphate (with water as the leaving group) to create an active imidoyl-phosphate. This would be in line with previously proposed mechanisms [117] and also congruent with the mechanism of urea participation in phosphonate ester hydrolysis [118].

Again, the synthesis of membrane making glycerol phosphate along with other reduced oxidation state phosphorus species by the reaction of Fe_3_P (major component of schreibersite) with glycerol under aqueous condition demonstrates the ability of such minerals in the prebiotic phosphorylation of biological molecules on early Earth [8].

**Figure 32:**
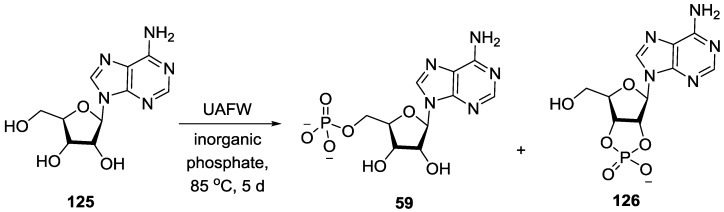
Phosphorylation of nucleosides in urea/ammonium formate/water (UAFW) eutectic solution as demonstrated by Burcar et al. [110].

As discussed elsewhere [4,119] minerals like schreibersite, hydroxyapatite etc. have been used in the prebiotic phosphorylation of nucleosides. Although the minerals phosphorylate the prebiological molecules, the major challenges associated with such phosphorylations are the low release of phosphates from such minerals. To deal with such low phosphate release, Burcar et al. used urea/ammonium formate/water (UAFW) based eutectic solvent mediated phosphorylation reaction, and demonstrated that the rate of phosphorylation was markedly increased compared to the use of urea alone for phosphorus minerals of varying solubility (Figure 32) [110]; the mechanism suggested here is in line with what has been proposed before with the oxygen of the urea attacking the phosphate to create an imidoyl-phosphate species [117]. Hydroxyapatite Ca_5_(PO_4_)_3_OH, one of the most common phosphorus minerals in Haden Earth, is insoluble in water but in the presence of this eutectic solvent is able to take part in the phosphorylation reaction with increased rate [120].

The more efficient mineral for prebiotic phosphorylation consideration is struvite (MgNH_4_PO_4_·6H_2_O). Pasek and Gull demonstrated the effectiveness of struvite in phosphorylating the prebiotic molecule like glycerol and choline chloride although they raised the question on the prebiotic relevance of struvite [121]. It has been shown previously that it was precipitated out when phosphate (PO_4_^3–^) was added to seawater containing more than 0.01 M ammonium [122]. But this precipitation requires high concentration of NH_4_^+^ ions [123], and its availability on early earth has been questionable [124]. The formation of struvite took place via the following reaction:

(1) $$Mg^{2+}+NH_{4}{}^{+}+PO_{4}{}^{3-}+6H_{2}O\overset{\;}{\to }MgNH_{4}PO_{4}\cdot 6H_{2}O$$

**Figure 33:**
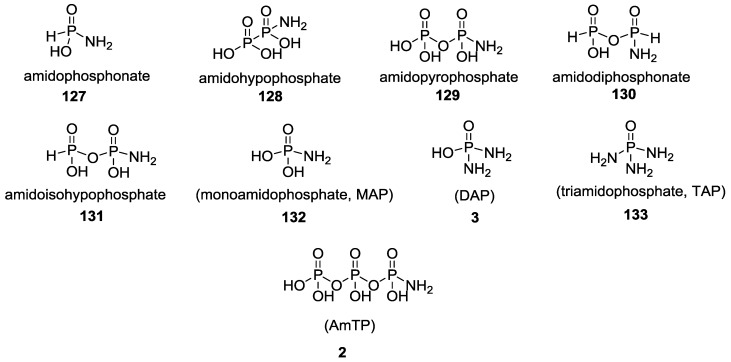
Plausible prebiotic nitrogenous analogues of inorganic phosphates.

As many of the above examples illustrate, there are many nitrogen containing compounds (such ammonium ions, urea, imidazoles and formamide) that have been invoked to enable phosphorylation in a prebiotic context. Extending this trend backwards in time, it is worthwhile to speculate about the existence and availability of the nitrogenous versions of the reduced phosphate species (as shown in Figure 33) derived from primordial P–N species [102,104,105,106,107]. This speculation also leads to the next question as to how these could be formed and what would be the environments, sources and reagents that would make them available in plausible prebiotic geochemical settings. Taking two of the species, AmTP **2** and DAP **3** in Figure 33, as examples we can make the following statements: 1) they have been demonstrated to be reactive and efficient phosphorylating agents of sugar molecules in water under plausible prebiotic conditions; and 2) they have been produced by the reaction of two plausible prebiotic source molecules, ammonia with trimetaphosphate (a cyclic condensed phosphate).

We propose that such reasoning and experimental approaches could be extended to the rest of the family of P–N compounds depicted in Figure 33, that not only include the phosphate versions, but also the reduced P versions of prebiotically plausible phosphonic acids and the corrosion products of minerals such as schreibersite (in meteorites) and related phosphides on Earth [125]. The challenge would be to detect or show that such nitrogenous versions did or could have existed or be generated on early earth, which would become important as the chemistries of these P-N derivatives continue to demonstrate their relevance to prebiotic chemistry.

In this context, it is once again important to note that the only known formation of AmTP **2** and DAP **3** have been demonstrated from TMP **1**, which itself is thought to be scarce on early earth. High concentrations of ammonia (in the 5–12 M ranges) were used with the sole purpose of driving the reaction towards complete formation of AmTP and DAP quickly. Such short-time conversions need not be necessary in the case of prebiotic geochemical scenarios, and could allow for lower milli- or micro-molar concentrations of compounds to react over longer time scales. Whether such concentrations of ammonia would have been available on early earth has been a matter of debate [126] in terms of a reducing atmosphere which is important for generation of organic compounds. However, there seems to be various avenues by which ammonia could have been available on early earth for further processing [127,128,129,130,131]. There are also some mechanisms that can provide some concentration of ammonia (as ammonium species) on early earth [132,133]. What this points out is also the need to search for alternative chemistries—coupled with other sources of phosphorus (such as the corrosion of reduced P species on early earth, for example, roaldite [134] which can co-occur with schreibersite, or reaction of phosphine [135,136,137] and/or other polyphosphate sources)—which could lead to reactive P–N derivatives (similar to AmTP and DAP), and would be a productive direction of future research activities. Given the lack of data regarding P–N species in terms of early earth geochemistry, it may be premature to speculate as to the type of geological setting where such reactions may take place; however, it may also present opportunities and new venues for geochemists to explore further.

## 11. Conclusions

The various chemistries of P–N derivatives remain largely unexplored and underutilized in the context of prebiotic phosphorylation chemistry even though they have been repeatedly demonstrated to function effectively in aqueous medium with respect to the important process of phosphorylation, overcoming the “water problem”. The examples reviewed above, drawn from synthetic organic chemistry and biochemistry show that P–N derivatives do have the capacity to phosphorylate a wide variety of substrates, and in many cases also have been demonstrated to work under prebiotically relevant aqueous conditions. Furthermore, some of these examples show the potential to go beyond the phosphorylation reactions. For example, amino acids once phosphorylated and having formed P–N derivatives have been shown to form peptides. These reinforce the possibility that there is not only potential for overcoming the phosphorylation problem, but also the potential to connect the various classes of compounds to their respective higher order structures (polymers). This could be extended to a) the nucleosides giving rise to oligonucleotides, and b) the formation of phospholipids giving rise to self-assembled enclosed structures such as vesicles. As more and more chemistries of the P–N derivatives are demonstrated in a prebiotically relevant context, the search for prebiotically plausible sources and existence of P–N derivatives on the early earth will become critical, if these findings are to have any meaningful impact on, and be pertinent to, the origins of life research.
